# Silicon Carbide Converters and MEMS Devices for High-temperature Power Electronics: A Critical Review

**DOI:** 10.3390/mi10060406

**Published:** 2019-06-19

**Authors:** Xiaorui Guo, Qian Xun, Zuxin Li, Shuxin Du

**Affiliations:** 1 School of Engineering, Huzhou University, Erhuan Road 759, Huzhou, China; guoxr@zjhu.edu.cn (X.G.); XQ09086320@163.com (Z.L.); xunq520@hotmail.com (S.D.); 2 Department of Electrical Engineering, Chalmers University of Technology, 313000 Göteborg, Sweden

**Keywords:** power electronics, high-temperature converters, MEMS devices, SiC power electronic devices

## Abstract

The significant advance of power electronics in today’s market is calling for high-performance power conversion systems and MEMS devices that can operate reliably in harsh environments, such as high working temperature. Silicon-carbide (SiC) power electronic devices are featured by the high junction temperature, low power losses, and excellent thermal stability, and thus are attractive to converters and MEMS devices applied in a high-temperature environment. This paper conducts an overview of high-temperature power electronics, with a focus on high-temperature converters and MEMS devices. The critical components, namely SiC power devices and modules, gate drives, and passive components, are introduced and comparatively analyzed regarding composition material, physical structure, and packaging technology. Then, the research and development directions of SiC-based high-temperature converters in the fields of motor drives, rectifier units, DC–DC converters are discussed, as well as MEMS devices. Finally, the existing technical challenges facing high-temperature power electronics are identified, including gate drives, current measurement, parameters matching between each component, and packaging technology.

## 1. Introduction

Power conversion systems are widely employed in the industry ranging from aircraft, automotive, deep oil/ gas extraction, and space exploration where high-temperature power electronics are required [1,2,3]. The aircraft field is moving towards more electric aircraft with the reduction or removal of the hydraulic, mechanical and pneumatic power systems [4]. This means more electrical actuators are needed to improve the efficiency, reliability, and maintainability. Power electronic devices, such as sensors and actuators should be placed close enough to the hot engine. Among them, some need to experience the ambient temperature varying from −55 to 225 °C due to the short distance to the jet engine, and the gas turbine must operate above 350 °C [5]. In electric automotive applications, operating ambient temperature ranges from −40 °C to a very high temperature differing with various locations. For example, the coolant temperature can reach up to 120 °C at 1.4 bar, the temperatures for wheel sensor and transmission are around 150 to 200 °C, and the exhaust sensor is up to 850 °C with an ambient temperature of 300 °C [6]. In the deep oil/gas extraction, the electrical downhole gas compressor is designed to improve the throughput of gas wells. The ambient temperature is expected to reach 150 °C since the compressor should be installed close to the gas reservoir, and the system is expected to work reliably under an ambient temperature of 225 °C with the lifetime of 5 years [7]. Regarding space exploration, it is obviously a “niche” market, but it is quite challenging to develop power electronics used for this application. The surface temperature on Venus can reach up to ~460–480 °C, while on Jupiter the temperature increases with depth and pressure [8,9]. The ambient temperature reaches 400 °C at 100 bar and comes along with a very aggressive atmosphere with wind speed around 200 m/s and hydrogen-rich chemical composition. Moreover, thermal cycling can be another challenge in the space since the ambient temperature is −140 °C during the night. In these applications, it is common that electronic equipment works in a quite harsh environment, especially an extensive temperature range, and frequent deep thermal cycling [10]. Table 1 summarizes the applications requiring high-temperature power electronics in current/ future technologies. 

To be able to withstand the high-temperature environment, power electronic equipment is always designed to have active or passive cooling systems [11]. These cooling solutions include forced convection air cooled heat sink, sided cooling with liquid cold plate, micro-channel liquid cooler built into the power module, in addition to jet impingement and direct contact liquid cooling. Although large numbers of thermal management solutions have been designed to cool the power electronics and manipulate their operating temperature, these cooling solutions in oil/gas extraction applications are not efficient or effective. Besides, those applications have the typical issue associated with obviously undesired high cost, extra weight, and volume by introducing the cooling system. Moreover, due to the limited information about the actual operating environment and the actual load cycling, there is a lack of accurate thermal analysis and reliability assessment for each component [12]. Thus, failure of the cooling system can readily jeopardize and even destroy the whole electronic system. Considering these factors, it would be appealing to have electronic components capable of enduring elevated and fast varying temperatures. As a result, system reliability can be largely improved while both the upfront and operating costs can be reduced. Accordingly, the development of high-temperature power electronics is of great importance and has attracted considerable research efforts. 

Currently, power electronic devices in conversion systems and micro-electromechanical systems (MEMS), such as DC/AC converters, AC/DC converters, DC/DC converters, control, ICs and sensors are mainly manufactured by silicon (Si) material [13]. The performance of Si-based power electronics has almost been driven to its logical boundary after about 60 years of tremendous development, but they still cannot offer satisfactory performance for many applications. For instance, the maximum permissible operating temperature for Si isolated gate bipolar transistor (IGBT) from Infineon is 125 °C, for Si MOSFET, the maximum obtainable junction temperature is 150 °C. Such a limited junction temperature together with switching frequency makes it unsuitable for individual application fields with required high-power, high-frequency, and high-voltage. To cater for the low-temperature tolerance, the Si IGBT is designed with complicated heat sink and maximum switching frequency at approximately 30 kHz. This structure results in cumbersome passive components, low power density, and poor dynamic performance. However, substantial improvement in power conversion systems is difficult with simple employment of fabrication technologies or Si semiconductor devices. The demerits of power electronics manufactured by Si materials are evident and thus limit industrial applications of power electronic devices [14].

Fortunately, the third-generation semiconductor materials, represented by silicon carbide (SiC) and gallium nitride (GaN), have gradually shown superior characteristics compared to Si material. Due to the wide band-gap, high breakdown field strength, high thermal conductivity, and fast electron saturation drift velocity, SiC is one of the most promising alternative materials and it is very suited for high-temperature power electronic devices [15]. Compared with conventional Si power electronic devices, commercially available SiC devices have not only better thermal stability and higher temperature tolerance but also lower switching/conduction loss. Consequently, SiC devices are quite a promising solution to converters tailored for high-temperature applications. In theory, the permissible junction temperature of SiC power electronic devices can reach as large as 600 °C, thanks to the wide band-gap of the semiconductor material, which is about three times of that of Si material [16]. The integrated power modules operating in the full temperature range are expected to be widely applied in the foreseeable future. 

Research on high-temperature power electronics is not new and has been going for 20 years ago. The first review paper on this research summarized many of the same challenges we are still suffering in today’s market [17]. The common conclusions have been drawn that the high-temperature power electronics cannot be developed by only one component. Although existing power converters equipped with SiC power electronic devices can operate at a top junction temperature above 150 °C [18,19], the heat-resistance attributions of high-temperature converters will disappear if gate drives cannot endure in a high-temperature environment. The associated issue for the gate drive is to be designed accordingly to match the requirement of high-temperature and high-speed capability. Furthermore, high-temperature passive components, such as capacitors, resistors, and magnetic materials, required to assemble high-temperature converters are equally as crucial as other components for reliable operation. The magnetic material can work in ambient temperatures up to 450 °C [20], but the issue comes for capacitors as most dielectric material cannot be used above 200 to 250 °C. Due to the performance of active and passive components which varies over the wide temperature range, the parameters matching should also be considered. Moreover, high-temperature packaging and integration technology are crucial for the design and development of high-temperature converters to give full play to high-temperature tolerance of SiC power electronic devices. Thereafter, there are some reviews on high-temperature power electronics, which are mainly focused on the process, packaging, ICs [21], MEMS sensors [22], and power converters [23,24], and the review on high-temperature applications is inclusive. 

In view of the above aspects, a comprehensive overview of the development profiles of high-temperature power electronics based on SiC converters and MEMS devices is conducted, however, has not been presented in the available literature. This work starts with some of the most prominent applications for high-temperature power electronics, fills such a gap by discussing state-of-the-art high-temperature components technologies, including SiC material, power devices or modules, gate drives, and passive components. It is identified that advanced material techniques are essential for high-temperature power electronics. Meanwhile, several exemplary applications of high-temperature power electronics, such as motor drives, rectifier units, DC–DC converters, and MEMS devices are analyzed, and the critical factors of performance promotion for converters are highlighted. Finally, the existing challenges in further advance of high-temperature power electronics are discussed.

## 2. High-Temperature Components

The electrical system, from power generation, power conversion, and power transmission to all kinds of power equipment, runs in a wide temperature range. However, these systems cannot be developed without the improvement of advanced material, power electronic devices, gate drives, and passive components [20]. This section outlines the development profiles of the high-temperature components. 

### 2.1. The Properties of SiC Material

In 1824, a Swedish scientist called J. J. Berzelius discovered the existence of SiC material, and subsequent research revealed that this material has good performance. However, SiC material had not been well-developed due to the outstanding achievement and rapid development of Si technology at that time. Until the 1990s, Si-based devices could not meet the high requirement of power electronics, such as high frequency, high voltage, high temperature, and high power density. This has once again ignited the interest of researchers in SiC material. 

Since the covalent bond between carbon and silicon is stronger than that between silicon atoms, SiC material have higher breakdown electric field strength, carrier saturation drift rate, thermal conductivity, and thermal stability compared to Si material. SiC material have a variety of different crystal structures (polytypes), and more than 250 have been identified to date. Although there are many types of polytypes, only three crystalline structures exist—cubic, hexagonal, and rhombohedral. The physical properties of the current available semiconductor materials are listed as Table 2. Despite the same atomic composition in all SiC polytypes, the electrical properties differ. For instance, the band-gap for SiC ranges from 2.2 eV for 3C-SiC to 3.2 eV for 4H-SiC. Since 4H-SiC has higher electron mobility than 6H-SiC, it is a preferable option for SiC-based devices. Due to that the thermal conductivity of SiC, which is three times that of Si, and it is expected to withstand higher operating temperature for devices equipped with SiC material.

However, high-purity SiC powder, which can be used to grow SiC boules, is only available from a limited number of suppliers, and is relatively expensive [25]. At present, the United States is the global leader in the production of SiC substrates and wafers, followed by Europe and Japan. The quality of SiC substrate is critical for the manufacturing of high-quality chips, and the SiC substrate constitutes a major portion of the chip cost. However, the cost of epi-growth and chips can also be reduced by the use of larger-area substrates, so manufacturers that are able to successfully fabricate 6-inch diameter SiC substrates with acceptable quality. From the Yole’s report, the market size of SiC N-type wafers will increase to US$110 million by 2020 with a 21% compound annual growth rate (CAGR). With a fast growing rate of CAGR, the production of SiC-based devices will be dramatically increased.

### 2.2. SiC-based Power Electronic Devices

(1) Development of SiC devices

**Figure 1:**
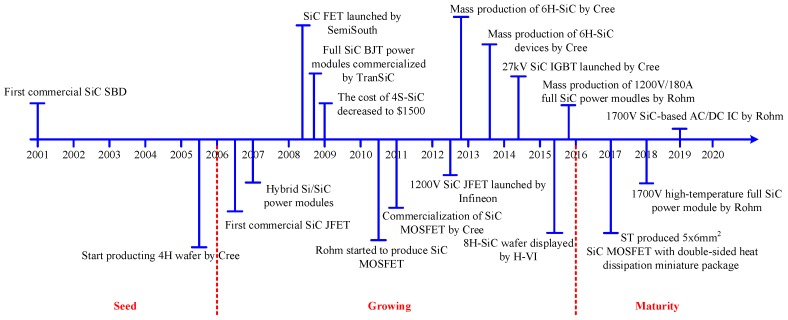
The milestones of the development process of SiC power electronic devices.

As early as 2001, Infineon produced the first commercial SiC Schottky barrier diode (SiC SBD) with characteristics of high blocking voltage, better thermal stability, and hardly any reverse recovery time. This paved the way for the development of SiC power devices in the field of power electronics. Since then, more discrete devices and power modules have gradually come out [26]. Figure 1 shows the milestones of the development process of commercialized SiC semiconductor devices. Until 2014, GeneSiC and Micross components have sold SiC bipolar junction transistor (BJT) with junction temperature up to 210 °C. At the research and development level, the operating junction temperature of SiC-SBD can reach up to 300 °C, and the performance of SiC positive-negative (P-N) diode under the temperature of 600 °C has also been verified. 

As the most market-oriented SiC devices at this stage, SiC MOSFETs have a fast switching speed and low on-resistance. In 1987, Palmour et al. from NCSU in USA developed the world’s first high-temperature depletion layer N-channel MOSFET. Subsequently, Brown et al. from GE integrated a simulated operational amplifier (OPA) using depletion MOSFET, and it can work at 300 °C. In 2011, Purdue University reported a SiC CMOS digital integrated circuit with the maximum temperature of 350 °C, but as the temperature continues to increase, the gate leakage current will increase rapidly. Studies have shown that the long-term reliability of the gate oxide structure of SiC MOS is not good, especially at high temperature, this issue is exacerbated. At present, commercial SiC MOSFETs can operate up to 200 °C [27]. 

**Figure 2:**
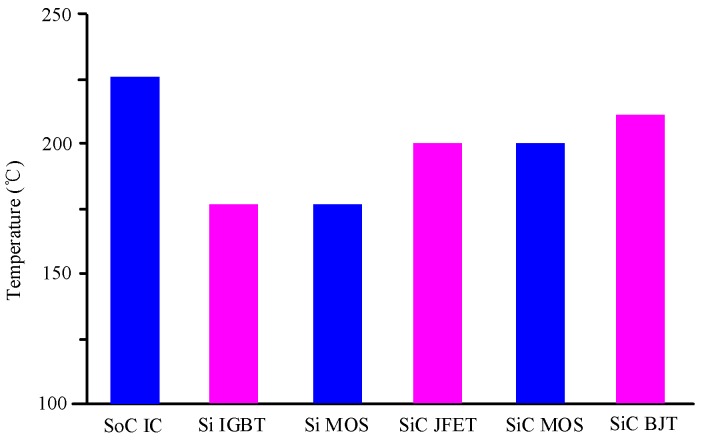
The maximum operating temperature of SiC power electronic devices.

Unlike SiC MOSFETs, SiC BJTs, with high reliability, are very suitable for high-temperature conditions. However, the disadvantage is that a continuous and stable driving current is required to cause a large loss, and the current gain decreases as the temperature increases, and then the driving loss is further increased. The commercialized 1,200 V SiC BJT produced by GeneSiC can withstand temperature up to 210 °C, which is the highest level in the market. Actually, SiC junction field-effect transistor (JFETs) has developed since the 1990s, and the first commercial SiC JFETs came out around 2006. In general, a lateral channel structure or a vertical trench structure is employed in a SiC JFETs. It shows that Infineon uses lateral channels, while Semi South mainly uses vertical channels. SiC JFETs produced by Semi South and packaged by Micross are resistant to temperature up to 200 °C. SiC JFETs is currently being studied by NASA Glenn Research Center, Rutgers University and Caesar Western Reserve University, and it is reported that SiC JFETs can operate reliably for 521 h at 460 °C. In 2016, NASS reported that SiC JFETs can operate for 25 h at 727 °C. Figure 2 compares the highest tolerated temperature for commercial power electronic devices at the current stage [28]. The advanced semiconductor materials are becoming the new choice for high-temperature power electronics. 

Theoretically, SiC devices, with wide band-gap, can allow a very high voltage and high operating temperature. However, the thermal capability of all materials has not reached the same technological maturity. The maximum operating junction temperature for most commercial SiC devices is only up to 210 °C. Tennessee University has developed the 1.2 kV/100 A SiC JFET power module operating at 200 °C. In [29], a 1.2 kV/60 A SiC MOSFET phase-leg power module with the optimized internal layout is presented for an operating frequency of 100 kHz and junction temperature of 200 °C. In [30], a SiC power module with a junction temperature of 250 °C is presented for military hybrid electric vehicle applications, which is designed as half or full bridge structure. Some discrete devices and ICs are demonstrated laboratory level to operate above 500 °C for a short while. Reference [31] shows the characteristics of MOSFET fabricated on β-SiC thin films, which can operate at the temperature of 650 °C. The research on material and fabrication of SiC devices is still ongoing to develop the high-temperature commercial SiC devices and modules. 

(2) Fabrication of SiC devices

**Figure 3:**
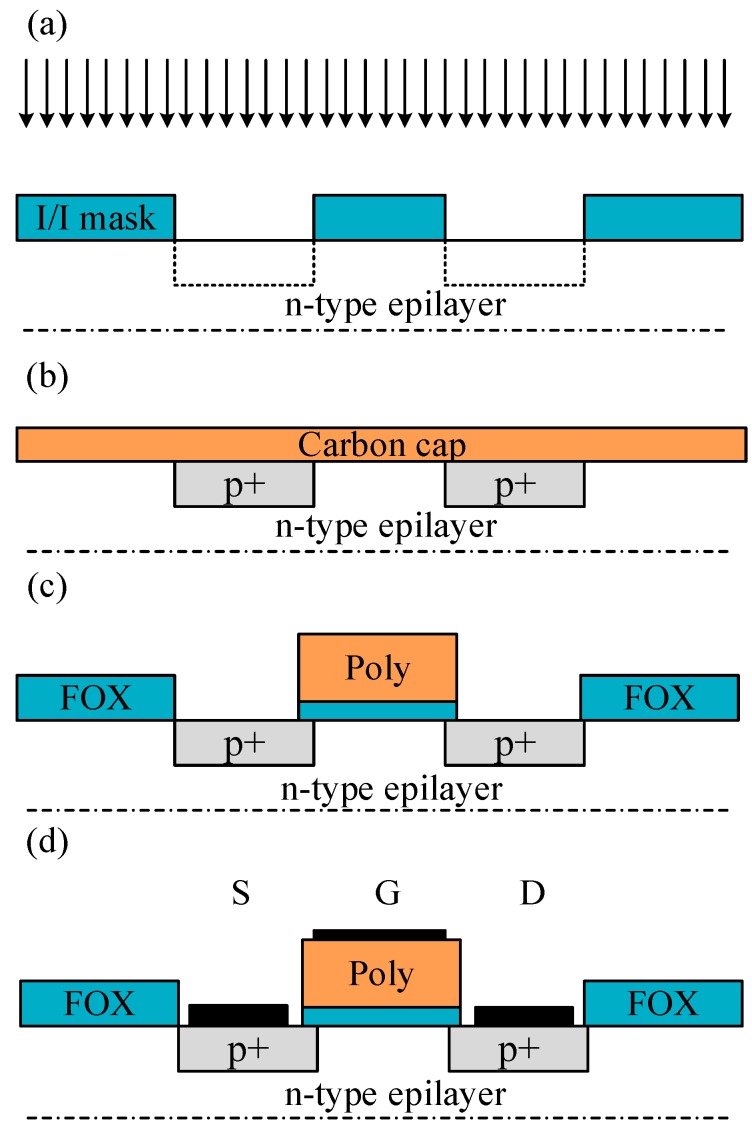
Simplified non-self-aligned SiC metal oxide MOSFET (**a**) Ion implantation of p-type dopants (arrows) into source and drain (dashed boxes) is performed with an ion implant hard mask; (**b**) during ion implantation annealing, the surface has to be protected with a carbon cap, which prevents Si out-diffusion and surface roughening even at 1,800 °C; (**c**) the field oxide (FOX) and polycrystalline silicon gate (poly) stack is aligned with global alignment marks (not shown), and some overlap must be allowed; (**d**) the metal must be patterned before annealing at ~800–900 °C to form the metal silicide (otherwise the reaction will occur with SiO_2_).

For an example of processing of SiC MOSFETs, wafer sizes and material quality for SiC have improved over time. The main difference between the processing of Si and SiC wafers is the temperature range, shown in Reference [32] for details. Since the strong bonds between silicon and carbon need more energy for the growth of material, post-annealing of damaged material after ion implantation, bond breaking during thermal oxidation or contact alloying. A simplified SiC MOSFET process flow in Figure 3 starts with ion implantation, field oxide formation, and polycrystalline silicon gate stack alignment. For SiC MOSFETs, the channel mobility improves slightly at higher temperature, and there is a maximum voltage rating that strictly limits the current drive and on-resistance. For ICs operating at high temperature, the condition will approach that of accelerated lifetime testing. Still, threshold voltages have to be designed with some safety margin at the highest temperature expected, where it is reduced by about 1 V with the rise of 100 °C.

At present, packaging technology is a big issue for design and fabrication of SiC power electronic devices or modules for high-temperature applications. Most packaging solutions are developed for mild ambient, and permissible junction temperature of SiC devices is far below the theoretical value. Packaging material and technology are critical factors for the further increase of the operating temperature. The future high-temperature converters require higher power density, which requires that the heat dissipation conditions would be as simple as possible, and the packaging technology should also adapt to the high-temperature situation. 

Ohmic contact and Schottky interface are critical factors that may limit the devices in the application of high temperature. High operating temperature could result in a diffusion process in the contact layer and a reaction between the contact components, which may result in changes in contact properties during high temperature operation, as well as degradation of the devices. In general, low contact resistivity should be maintained to decrease the voltage drop, but to get the low resistivity of ohmic contact is difficult for SiC devices due to the difficulty in doping and, in the case of p-type materials, due to the high electron affinity and high width of the band-gap. In addition, with the increase of temperature, Schottky barriers between metal and semiconductor become larger, which makes ohmic contact on SiC more difficult. By contrast, with Si and GaAs devices, the operating temperature is limited by the electronic properties of the semiconductor material; but the maximum operating temperature of SiC devices is limited by the stability of the contacts. Some device parameters such as response time and output power, depend strongly on the ohmic contact resistivity and its stability at high operating temperature. Therefore, the thermal stability of ohmic contacts and Schottky interfaces at high operating temperature is considered the critical factor for determining their power application.

**Figure 4:**
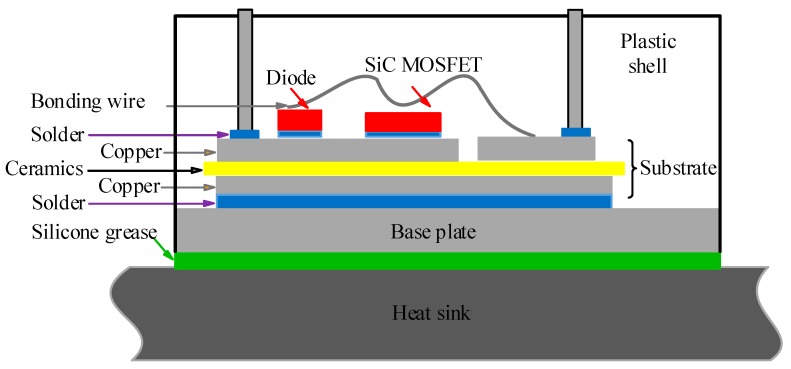
The package of SiC power module.

Device applications are presented as discrete devices and power modules, and wire-bonded package, shown in Figure 4, is commonly used in high-temperature converters. The substrate, base plate, die attach and heat sink are included, and the following will analyze the physical structure and composition material of SiC power module. 

● Substrate

The substrate, as the backing of SiC chips, must meet the material requirement with characteristics of excellent thermal conductivity, high mechanical strength, high flexural strength, and the similar coefficient of thermal expansion (CTE) with SiC. The direct-bond-copper (DBC) substrate presents a sandwich structure, which consists of two layers of copper, lying on the top and bottom, and with insulation ceramics in between. The available materials for insulating ceramics could be quantized as Al_2_O_3_, AlN, BeO, and Si_3_N_4_, and Table 3 shows the thermal mechanical characteristics. As it can be seen, the CTE of AlN is close to that of SiC material with the value of approximately 4–6 ppm/°C, and the mechanical stress resulted by heat expansion can be significantly reduced by the uniformity of parameters. Moreover, the thermal conductivity of AlN is highly relative to two other materials like Al_2_O_3_ and Si_3_N_4_, which will significantly promote the cooling capacity of power modules. Therefore, AlN is one of the most appropriate choices for the substrate to encapsulate SiC power modules, and the relevant power modules can endure in the high-temperature environment which is above 250 °C [33].

● Base plate

The base plate, as the foundation and heat conductor of power modules, also has the strict material requirement, which should have characteristics of excellent thermal conductivity and similar CTE with the substrate [34]. Table 4 shows the thermal mechanical attributes of possible materials for the base plate. CTE of Cu and Al are much higher than that of AlN (only 4.5 ppm/°C), while CTE of metal matrix composites with Mo and Cu is close to that of AlN. The thermal conductivity of metal matrix composites makes these materials have excellent heat dissipation property. With these advantages, the metal matrix composite is an excellent choice for power modules to endure in a high-temperature environment. Due to the adjustable CTE from 6.5 to 9.5 ppm/°C, and relatively high thermal conductivity with the range of 170–200 W/m·K, AlSiC is also an excellent material to constitute base plate.

● Die attach

Die attach, also known as die bonding, is the process of connecting or bonding a die or chip to a substrate, package or another die. This process can happen in many forms and can be applied in many ways, and differences between them are entirely dependent on the user’s desire. Various options are discussed in [20], including high lead solder alloys, lead-free solders, high-temperature reflow soldering, nano-silver paste sintering, and transient liquid-phase bonding. Transient liquid-phase bonding is capable of bonding at low temperature and servicing at high temperature, which is a significant trend in the field of high-temperature packaging for SiC power modules. Due to the disadvantages of time-consuming nature and required special equipment, some research on high-throughput solutions is under way [35].

Solder is required to have characteristics of a high melting point, strong bonding strength, excellent thermal conductivity, and electrical conductivity. It can efficiently conduct the heat generated by switching losses to a radiator in time. Thus, it dramatically improves the capabilities of SiC power modules to operate reliably under the condition of high frequency and high-temperature [36]. Some readily available lead-free alloys fall within the category of the high melting point, with melting points well above 220 °C, which are listed in Table 5. The lead-free alloys are a good option for high-temperature die attach. As can be seen, Au20Sn80 with the highest melting point of 280 °C is suitable for high-temperature soldering and packaging. Gold-based solders such as AuSn, AuGe, and AuSi, have a good reputation because of their melting points of 280 °C, 365 °C, 363 °C or so, respectively, but the gold-based solders are costly and not suitable for mass production. Other alloys such as zinc and bismuth alloys have been studied for their potential as well [37,38]. Silver sintering has better long-term reliability compared to leaded-free solders, but the challenge of microstructure evolving or coarsening at elevated temperature is a hindrance. 

● Heat sink

Generally, the heat sink is joined to the power module by silicone grease, and in this way, the heat generated by all kinds of losses dissipates to the air. The thermal resistance of silicone grease occupies 50% in the whole module, and thus thermal interface materials (TIM) become one of the critical factors to significantly reduce the radiator volume and increase converter power density [39]. Moreover, the grease will dry out and age as time goes on. Therefore, some innovative solutions to reduce the thermal resistance and optimize the power dissipation should be developed. 

TIM research has focused on three primary areas of using high-performance fillers, studying the micro and nanoscale TIM, and developing carbon-allotropes-based TIM to enhance performance [40,41,42]. Carbon nanotube fillers are the most promising of all the carbon allotropes, and have become front research topics in TIM which can decrease thermal stress caused by CTE mismatch and can keep chemicals table over a wide range of temperatures.

The high-performance cooling methods are jet impingement cooling, spray cooling, and micro channels, and all of them can be designed as two-phase flow with the increased heat transport capability [43,44]. A model of single and two-phase liquid cooling using micro channels was established for GaN-on-SiC field effect transistor (FET) [45]. Furthermore, an additional method is the direct-immersion cooling technique, where the coolant liquid is in direct contact with the power electronics device. However, the notable problems of coolant leakage and compatibility requirements result in preference for indirect cooling.

### 2.3. High-temperature Gate Drives 

In ambient temperatures above 150 °C, a real challenge is to seek a proper gate drive circuit to match with attributions of heat resistance and high frequency of SiC power electronic devices. In theory, the junction temperature of SiC power electronic devices can reach up to 600 °C, which are commercially available for high-temperature applications. However, the integrated drive circuits for SiC devices are still in an early stage of research and development.

**Figure 5:**
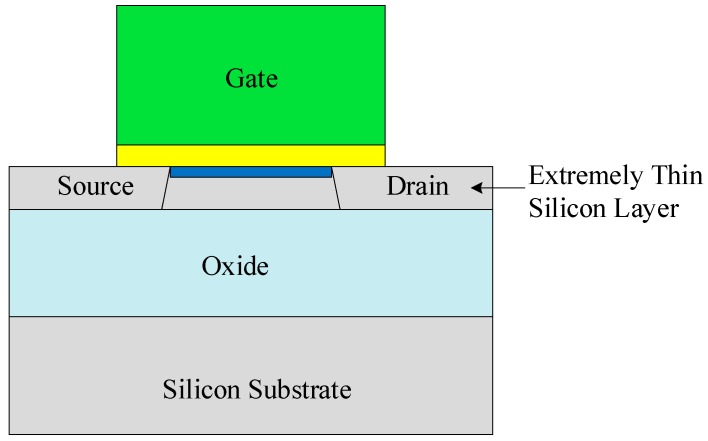
A buried insulator layer in a fully depleted SOI structure.

Based on the silicon-on-insulator (SOI) technology, a buried insulator layer in SOI structure shown in Figure 5 can effectively reduce the leakage current in high-temperature operation, improve the latch-up immunity, and suppress the threshold voltage variation to the temperature. The SOI-based integrated circuits can successfully operate at the temperature range from 200 °C to 300 °C [46], which is much higher than that of conventional bulk Si devices.

Compared to the SOI-based integrated circuit technology, the SiC-based integrated circuit technology is more attractive to extremely harsh environment [47]. However, the development of semiconductor technology presents many difficulties. Researchers have spent nearly half a century producing the first commercial SiC devices since the excellent characteristics of SiC material were discovered. However, the SiC-based integrated circuit remains at the stage of laboratory investigation, and is therefore unable to access the market in a short time. Regarding high-temperature gate drives of SiC devices, SiC-based integrated circuit technology is not mature enough yet, and Si-based integrated circuit technology is unavailable for high-temperature applications. Instead, Si-based discrete device technology may be a feasible solution when compensating temperature drift. In addition, the SOI-based integrated circuit is available for high-temperature applications when ignoring the high manufacturing cost [48].

High-temperature gate drives are required to have the input signal isolation, wide duty cycle range, static turn on/off, and low propagation delay time. Currently, no commercial opt couplers can be used in a high-temperature condition. As a result, the transformer becomes the best choice for high-temperature isolation of the gate drive. For a developed SiC MOSFET phase-leg power module, an input signal isolator is used to build an SOI gate drive chip. Even though SOI die, printed circuit board (PCB) and passive components can accommodate the high-temperature environment above 150 °C, but the SOI gate drive board cannot withstand such temperature. Its bottleneck is the commercial isolator, whose maximum operating temperature is 105 °C. Air-core transformer isolators for the SOI-based integrated circuit are under active study, with the hope to reduce the volume of natural chip-level integration and to eliminate the high-temperature ageing effects of magnetic materials [49]. 

As the SOI technology is well developed, companies, such as CISSOID, XREL, and Honeywell, have developed the high-temperature gate drive circuits. CISSOID provides an isolated gate drive circuit integrated with high-temperature SiC MOSFET devices in the shape of the intelligent power module (IPM), which dramatically reduces the effect of the parasitic parameters and allows the maximum ambient temperature up to 225 °C [50]. The gate drives manufactured by XREL can operate at the ambient temperature of 230 °C. The SOI technology can also be used to develop high-temperature drive ICs, but the manufacturing process is complicated and associated with the high cost. The gate drive ICs based on SiC technology can operate reliably at the temperature of 400 °C, and it has been reported in [51,52,53,54]. However, this technology is not mature and commercialized yet.

**Figure 6:**
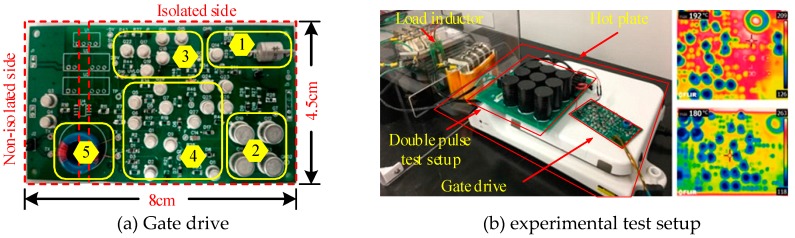
Developed high-temperature gate drive and the experimental set setup.

Due to the high cost of SOI ICs and SiC fabrication technologies, an alternative way to develop high-temperature gate drives is the utilization of commercial-off-the-shelf (COTS) discrete transistors and diodes. With the COTS discrete component, the operating temperature of gate drives can reach up to 180–200 °C [55]. The main drawback of this kind of gate drives is the large propagation delay due to the high number of SOI ICs, and the protection features of desaturation and under voltage lockout (UVLO) are not included. To solve these issues, a COTS gate drive is proposed with the integrated circuit of overcurrent and UVLO, which can operate under the ambient temperature of 180 °C [56]. The proposed COTS gate drive shows better performance than the commercial gate drive (EVK-HADES 1210) produced by CISSOID in the aspects of propagation delay and total power consumption. Figure 6 shows the prototype of the COTS gate drive and the test bench. In Figure 6a, the numbers shown from 1 to 5 represent on-state voltage monitoring diode, driving buffer stage, overcurrent detection circuit, UVLO with control logic, and isolated transformer, respectively. 

### 2.4. High-temperature Passive Components

(1) Capacitors

Capacitors are an irreplaceable component in the power electronics, and they need to be selected carefully for converters, since capacitance, insulation resistance, reliability, and service lifetime will be reduced to some extent with the increase of temperature [51]. The temperature dependency of these characteristics is related to capacitor structures and materials. Among various dielectric materials, only a few are available for high-temperature operation, such as ceramic, mica, and tantalum [57]. Mica-10 shows prominent characteristics even at 200 °C with an energy density of 11.27 J/cm^3^ and efficiency of 94.7% at 500 MV/m, which is 30 times higher than the well-known polymer PI for high-temperature applications [58]. The tantalum capacitor is one option for high-temperature applications, and it can reliably work at elevated temperatures above 200 °C [59]. Considering the low capacitance of the mica capacitor and low voltage ratings of the tantalum capacitor, the ceramic capacitor is a preferable option for high-voltage and high-power power electronics.

C0G and X7R ceramic capacitors are rated for high-temperature operation. The capacitance of C0G decreases by 1% when the temperature varies from 25 °C to 250 °C. Although C0G capacitors present excellent temperature stability, capacitances are too low to meet the requirement of the high-power system. For X7R capacitors, capacitance is in the range of microfarad, which is almost an order of magnitude larger than C0G capacitors. However, the X7R capacitors present wicked capacitance stability, and the capacitance will be reduced 42% from 25 °C to 250 °C coming along with an excessive capacitance variation with operating voltage [60]. Moreover, the insulation resistance is only 210 kΩ at 200 °C, which is far below the 3 GΩ of C0G capacitors. This makes X7R capacitors unsuitable for most signal processing applications. 

**Figure 7:**
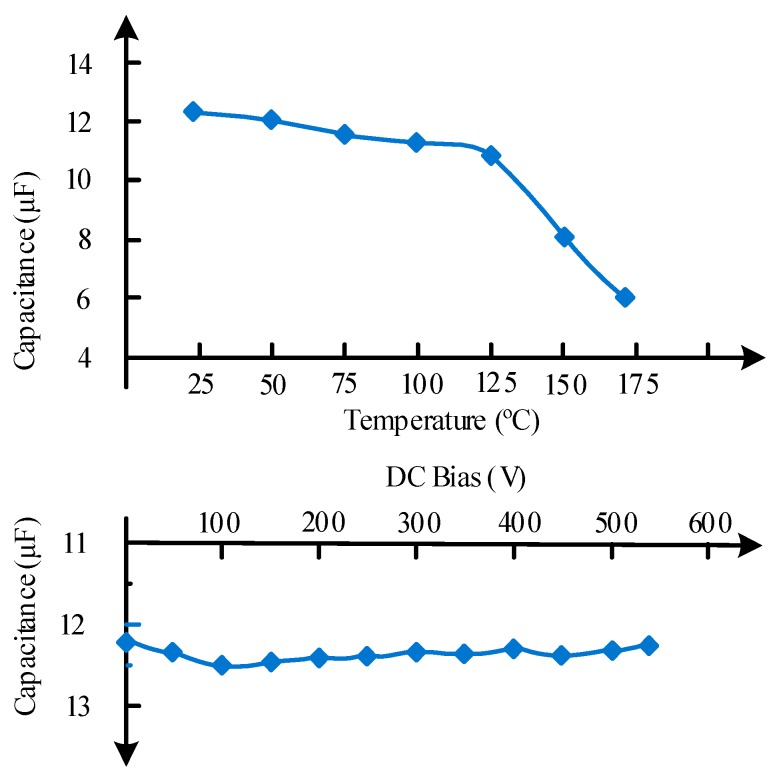
Capacitance stability with temperature and DC link voltage for the XHT stacked ceramic capacitors from Presidio.

Alternatively, the stacked ceramic capacitors with XHT dielectric from Presidio components, Inc. present improved capacitance stability when DC bus voltage is very high, as shown in Figure 7. A capacitance reduction of 50% from 25 °C to 175 °C is quantified. This kind of capacitors is more to cracks when used under the conditions of shock and vibration. Thus, the de-rating and reliability should be considered when designing capacitors for a high-temperature converter. With the new materials and fabrication technologies, Si capacitors exhibit the promising temperature resistance up to 250 °C [60]. Table 6 shows some commercially manufactured capacitors for high-temperature applications, and few comments are also made in this table.

(2) Resistors

Resistors represent the basic component providing protection, ballasting, sensing, feedback, and signal attenuation in power electronics. Signal processing is the main contribution of resistors, in addition to high reliability and stability, which are required in such systems. Resistor manufacturers have made great effort to develop high-temperature resistors. Table 7 summarizes some commercially manufactured resistors with some short comments [16].

Metal foil resistors have high precision and stability in harsh environments, but the maximum operating temperature is limited to 240 °C due to the bondable chip, wire-wound resistors show quite good high-temperature characteristics, but they are not suitable for high frequency, and they act as inductors at high frequency. Thin film resistors seem to be an economical way with small size and good performance, but they are not suitable in overload conditions, whereas thick film resistors show superior overload characteristics [61]. 

(3) Magnetic elements

For inductors and transformers, it is necessary to take into consideration the high-temperature characteristics and insulation properties of windings and magnetic cores. High-temperature-resistant enameled wires and Kapton electrical tape are readily available above 200 °C. As for the magnetic cores, high-temperature characteristics are affected by Curie temperature, coating materials, and power losses. The optimal operating point for most magnetic material is approximately 100 °C for most magnetic materials, and the efficiency will be reduced with the further rise of temperature.

The magnetic elements with rated operation temperature above 200 °C mainly involve three types of magnetic cores such as ferrite cores, power cores, tape wound, and cut cores, which are usually utilized for power supply, signal isolation and current detection in high-temperature converters. However, the operating temperature will affect their magnetic properties, and the B/H curve will drift with the temperature variation, which will lead to a significant detection error, especially for measurement sensors. Therefore, a compensation algorithm is needed to improve the detection precision. Reference [62] shows a strong correlation between the temperature variation and the magnetic characteristics of Mn–Zn Ferrite materials, the magnetic characteristics should be assessed when these components work in such thermal conditions. Ferrite cores with Curie temperature of 350 °C have been commercially available so far, and they can work reliably up to 250 °C.

## 3. High-Temperature Converter and MEMS Devices

With the commercialized development and mature application of SiC power electronic devices, high-temperature converters and MEMS devices have brought and will bring broad prospects in electric vehicles, more electric aircrafts, deep-earth oil explorations, and geothermal energy exploitations. 

### 3.1. SiC-based Motor Drive

High-temperature motor drives are required for the flight control actuators and fuel pumps of the aviation system as well as for the control units of electric vehicles, which will face the challenge to meet the requirement of higher efficiency, higher power density, and higher reliability in harsh operation condition. Take an example of a new generation of high-power electric vehicles; the motor drive system will undergo a change from traditional industrial grade to automobile industrial grade. United States Department of Energy draws up the development goal for hybrid electric vehicle (HEV) by 2020, the dramatic advancement will be developed. The power density of power electronic equipment will be higher than 14.1 kW/kg, and volume will be less than 13.4 kW/L, efficiency will be higher than 98%, the price will be lower than 3.3 $/kW [63]. SiC power electronic devices make motor drives have the ability of high-temperature operation, which plays an essential role in goal achievement.

**Figure 8:**
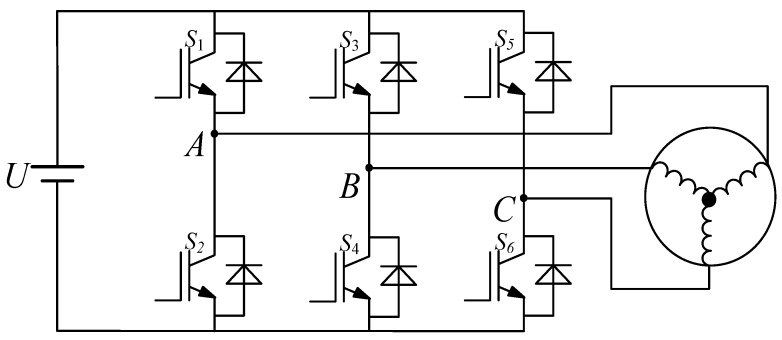
Electrical connection diagram of three-phase inverter and motor.

Figure 8 shows the electrical connection diagram of a three-phase inverter and motor, where a selection of power electronic devices is full SiC-MOSFET, full SiC-JFET, full SiC-BJT, or SiC/Si (e.g., SiC-SBD/Si-IGBT) hybrid devices. As the anti-parallel diode, SiC-SBD is hardly any reverse recovery time and is not affected by temperature variation. The radiator volume of a 2.5 kW motor drive based on SiC-SBD hybrid devices is reduced by 2/3 when compared with the radiator of the motor drive based on Si diode [64]. In [65], a three-phase air-cooling inverter is designed by using full SiC-JFET devices, the motor drive can work in the high-temperature environment of 200 °C, and the output efficiency with 18 kW power rating reaches up to 98.2%.

**Figure 9:**
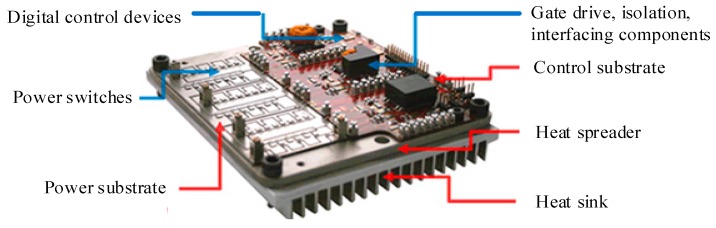
Multichip power module with high-density power electronics designed by Arkansas Power Electronics International.

**Figure 10:**
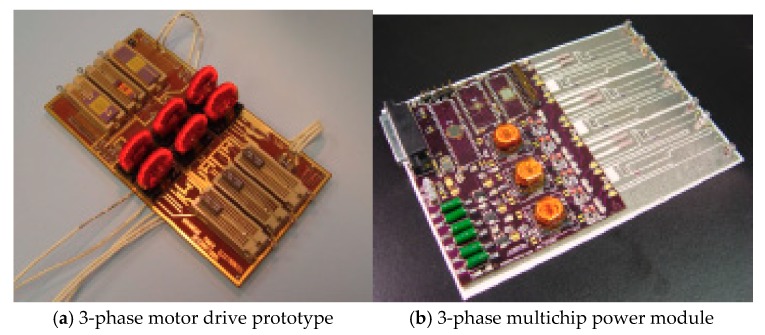
A prototype of 3-phase motor drive by using multichip power module (MCPM).

APEI Company has developed a 4 kW high-temperature motor drive by using the SiC-JFET multichip power module (MCPM) and SOI-based integrated circuit technology. The core of control electronics is a high-temperature microcontroller (SOI-based MOS HT83C51) produced by Honeywell Inc., outputting low power signals. Thus, an amplifying circuit provides a connection between the microcontroller and the power module, and the amplifying circuit is constituted of the high-temperature operational amplifiers and high-temperature passive components. A high-temperature transformer is designed by APEI to isolate the digital controlled circuit from the power circuit. Figure 9 shows the mentioned MCPM with high-density power electronics designed by APEI [66]. The MCPM approach is one of the packing strategies for power electronics, and the main idea is that the control and power circuitry components are integrated together into a single compact power module. AlN DBC material for power substrate and AlSiC material for heat spreader connected to the DBC substrate are selectable due to their excellent thermal conduction capabilities and a close CTE match. Multilayer polyimide for PCB is available for high-temperature applications since the glass transition temperature (Tg) is as high as 260 °C. An implementation of MCPM in a 4 kW 3-phase motor drive shows the SiC-JFET can operate at a junction temperature of 250 °C, Figure 10 shows the prototype of the 3-phase motor drive and the 3-phase multichip power module. Based on these state-of-the-art technologies, the motor drive module can operate in a high-temperature environment. Meanwhile, the converter has a substantial increase in respect of efficiency and power density.

### 3.2. SiC-based Rectifier Unit

The DC secondary power source is necessary for more electric aircraft in which the main power supply is an AC power source [67]. Here, the transformer rectifier or three-phase AC–DC converter can be used for rectifying 115 V AC bus voltage into 270 V DC, which supplies power to nitrogen generator system, engine starting system, environmental control compressor, hydraulic pump and other loads [68]. In a diode rectifier circuit, SiC-SBD with little reverse recovery current is used to reduce the switching losses, heat radiation requirements and high-temperature limitations for aviation rectifier unit. NATO is planning that SiC power electronic devices are applied to meet needs for converters to operate in a high-temperature environment in all-electric combat vehicles. Then, a 30 kW rectifier bridge is designed for the automobile application with 200 °C operation temperature. 

**Figure 11:**
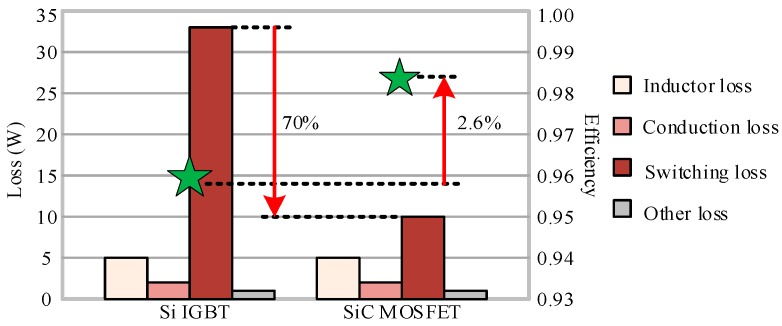
Loss and efficiency comparison of the rectifier with Si IGBT and SiC MOSFET.

For large-power high-temperature converters, the efficiency requirement is especially crucial except for reliability requirement in a harsh environment. The high-temperature converter with SiC power electronic devices can have a significant increase in efficiency due to the substantial reduction of switching losses. Figure 11 shows the main types of losses in a voltage doubling rectifying circuit and the efficiency comparison of the rectifier with Si IGBT and SiC MOSFET in same voltage level and control mode. The efficiency of SiC devices rectifier is 2.6% higher than that of rectifier equipped with Si devices. In addition, superior electric characteristics with low on-resistance at high-temperature and simultaneous high-speed switching conditions are also come with SiC devices rectifier.

**Figure 12:**
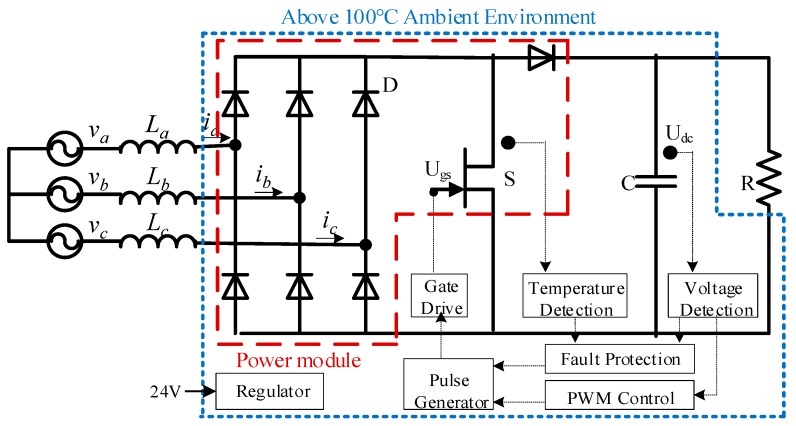
Hardware configuration diagram of the high-temperature three-phase AC–DC converter.

Figure 12 shows the hardware configuration diagram of the high-temperature three-phase AC–DC converter with the integration of 1 SiC-JFET and 7 SiC-SBDs, which can be applied to more electric aircrafts with the 270 V DC output, and it can also operate reliably in 200 °C ambient environments. Each component in the rectifier system should be carefully designed. Regarding SiC power modules, the mismatch of parasitic parameters, the variance of device property, and nonidentity of junction temperature can result in the unbalanced electro-thermal stress. The imbalance of multichip in parallel can be reduced by optimizing the layout of a direct bonding base, adding the low series resistance and coupling inductance, designing the feedback control of the drive circuit [69]. Here, the SiC power module using the flat-packaged structure has advantages of small parasitic parameters, flexible line layout and, double side cooling characteristics. In switching individual tests, the parasitic inductance is reduced by 14 nH compared to the pin-packaged structure, which makes the drain-source spike voltage decrease from 295.7 V to 279 V and further lowers switching losses [70]. In temperature detection, the temperature rise of grid resistance and the voltage regulator is apparent, but the temperature rise is not more than 10 °C from the environment temperature, which benefits from the lower power losses and excellent heat-sinking capability of the high-temperature AC–DC converter.

**Figure 13:**
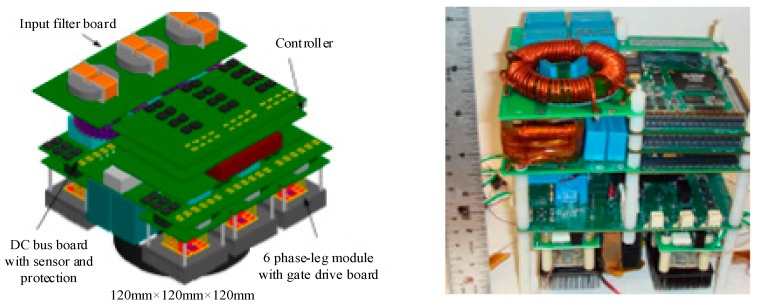
Conceptual drawing and the assembled prototype rectifier system.

In [71], Virginia Tech developed a 15 kW 650 V dc/230 V ac three-phase rectifier with interleaving structure by substitution of all Si devices with SiC JFETs, and the SiC power modules in the rectifier can operate at the junction temperature of 250 °C. With the volumetric power density of 6.3 kW/L, it successfully achieved a 2 kW/L target in more electric aircraft. It gives a detailed design for each component, including the active component, passive component, and the system. Figure 13 shows the drawing and the prototype of the rectifier system. 

### 3.3. SiC-based DC–DC Converter

DC–DC converters are widely used in the electric vehicle, more electric aircraft, and renewable energy. For example, high power HEV, the electric drive system is made up of a storage battery, DC–DC converter, inverter, electric machine, and control circuit, in which DC–DC converter plays a role in boosting the DC voltage for the post inverter unit. The permissible operating temperature for main components in HEV is listed as 120 °C for the motor, 200 °C for a turbocharger, 200 °C for throttle, 145 °C for the gearbox, 175 °C for driving chain, and 650 °C for exhaust pipe [72]. Due to the electrification and electromechanical integration of HEV, as well as the limitation of self-cooling capacity, these harsh conditions give power converters characteristics of reliability when operating in a high-temperature environment. Another example is when the DC–DC converter is applied to the generator control unit (GCU) in more electric aircrafts. The converter provides different levels of DC voltage for the various functional modules of GCU. The high voltage levels reach up to tens of thousands of volts, which are mainly used for communication, radars, transmitters of electronic warfare equipment, and a variety of cathode ray tube displays. The low voltage levels are classified as 24 VDC, ±12 VDC, ±5 VDC, ±6.3 VDC, and ±3.3 VDC. Due to the development tendency of the control system from traditional centralized engine mode to the distributed mode, the GCU would be placed closer to the generator, which makes converters operate in a harsh environment (– 55 to + 200 °C).

**Figure 14:**
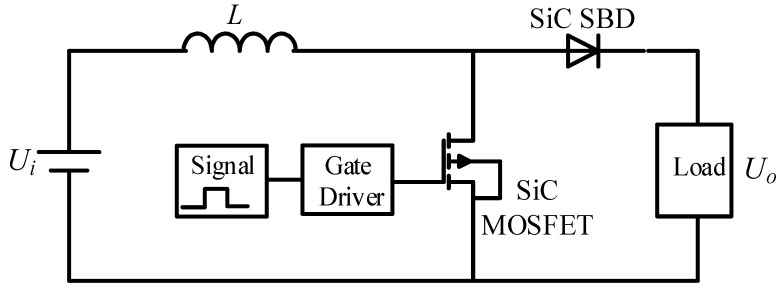
A topology of the boost DC–DC converter.

Figure 14 shows the circuit diagram of the boost DC–DC converter with SiC-MOSFET and SiC-SBD. Usually, Si-MOSFET has a large junction capacitance, which defines the switching frequency up to 100 kHz. Si-IGBT has current lag which limits the switching frequency up to 30 kHz. SiC-MOSFET can operate at a switching frequency of 200 kHz, or even MHz-level [73]. 

**Figure 15:**
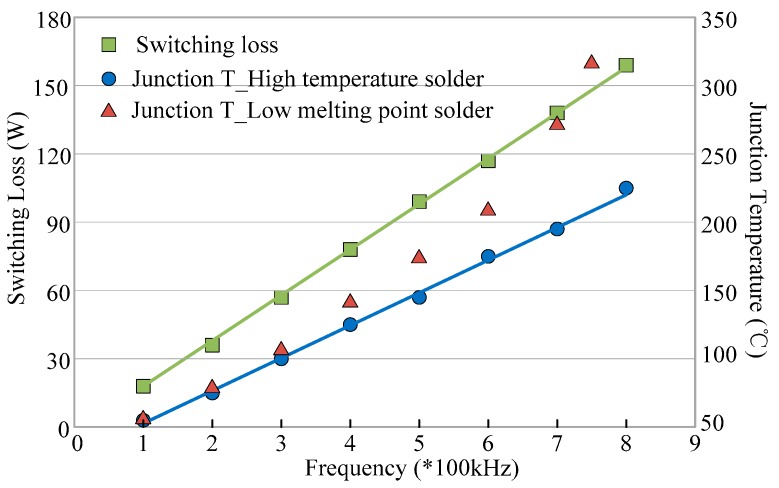
Switching loss and junction temperature distribution diagram under different frequencies.

For Si power electronic devices, switching losses increase significantly with the increase of switching frequency. When it mentions SiC power electronic devices, the switching losses, and junction temperature are measured as shown in Figure 15, where the switching frequency of SiC-MOSFET changes from 100 kHz to 800 kHz in a 1 kW boost DC–DC converter [74]. It can be seen that both switching loss and junction temperature keep a linear relationship with switching frequency when the high-temperature and thinner layer solder is used for die attach. If the solder with a low melting point of 180 °C and low thermal conductivity is adopted for the die attach, the junction temperature can rise at an accelerating rate when the switching frequency is above 500 kHz. NASA has reported that a 100 kW DC/DC converter based SiC JFET can reach up to the operating temperature of 415 °C in 2006. In 2008, Mazumder [75] reported that the efficiency of a DC/DC converter based on SiC-JFETs can reach up to 95% at 20 °C, while the efficiency of 100 V/270 V 2 kW boost converter proposed by Kosai [76] can reach up to 90% at the temperature 200 °C, the design and performance of the boost converter were evaluated over the temperature range from 20 °C to 200 °C. The capacitance variation of the output filter is also presented in [74], reporting that a 1 kW all-SiC boost converter with the output voltage of 800V can work reliably over a switching frequency range of 100 to 800 kHz, and the steady-state working junction temperature of SiC MOSFETs has been extended to 320 °C. However, the high-frequency gate drive capability and high-temperature die-attachment technology can be the issues to develop SiC-based converter operating beyond 320 °C junction temperature.

### 3.4. SiC-based MEMS Devices

The excellent mechanical properties of SiC material, coupled with the good thermal stability at high operating temperature, offer new possibilities for developing MEMS devices for extremely harsh applications compared to those possible with Si devices. 

The measurement and control technologies are also required for the high-temperature converters, and SiC devices allow the functionally integrated circuits (ICs) to operate in extreme environments. Many researchers have been working on the high-temperature ICs implemented by using SiC CMOS, JFET, and BJT, and developing the digital logic circuits, operational amplifiers, and memories. Reference [21] demonstrates that SiC makes high-temperature electronics possible up to 600 °C, reviewed the current technology performance and processing challenges relating to making ICs in SiC, and addressed that SiC devices should be commercially available in increasing quantities going forward, although the technology choice is unclear.

**Figure 16:**
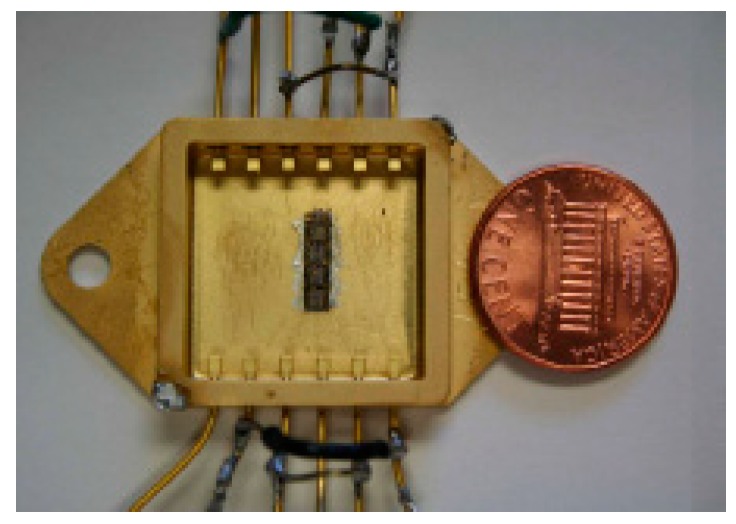
An optical photo for 4H-SiC power integrated circuit after packaging.

The first SiC-based power ICs were reported in 2008 [77]. Figure 16 shows the optical photo for 4H-SiC power integrated circuit after packaging, which includes a large power JFET and two buffer circuits. Reference [78] proposes an integrated bipolar OR/NOR gate based on 4H-SiC BJTs, and it can successfully operate up to 500 °C. References [79] and [80] report the differential amplifiers based on 4H-SiC JFET and 6H-SiC bipolar can reach up to the temperature of 500 °C and 600 °C, respectively. In [81], a 500 °C Schmitt trigger in 4H-SiC has designed and characterized, the proposed Schmitt trigger shows superior characteristics with a higher slew rate and almost independent temperature operation. 

A linear voltage regulator based on the nMOS SiC has been successfully designed and tested under at 300 °C [82]. In [83], a bipolar SiC linear voltage regulator was developed to operate at 500 °C. Regarding the ICs structure, in [84], the authors propose a novel 4H-SiC lateral BJT design with symmetric and self-aligned structure, the simulation, and optimization are conducted to operate at the temperature range of 27–500 °C with an optimal current gain. is the study demonstrates that the self-aligned 4H-SiC lateral BJTs design is easier and less costly to produce, with >90% smaller than a conventional structure. 

A monolithic SiC drive circuit for SiC BJTs was designed by Kargarrazi et al [85]. The performance was tested using a commercial power BJT under the resistive and capacitive conditions with the operating switching frequency up to 500 kHz. The SiC drive circuit has a good robust capability to the temperature range from 25 °C to 500 °C. In their latest publication, the controlled duty cycles from 0.5 to 0.7 are demonstrated with the operating frequency ranges from 160 to 210 kHz [86]. 

MEMS switches have mainly been developed in a broad swath of RF and microwave applications, and they could possibly replace positive-intrinsic-negative (PIN) diode, mechanical, FET, and other types of switches [87]. When compared to traditional micromechanical switches, MEMS switches have several advantages, such as lower insertion loss, higher isolation, and better switching figure-of-merit. They are widely used to measure oil pressure, fuel pressure and tire pressure in automotive applications, electronics, and telecom. The Foxboro is the first company who is involved in MEMS switches with the invention of the first electromechanical switch patent in the world in 1984. Analog Devices, Inc. has been started the research of MEMS switches since 1990, with the first MEMS accelerometer product successfully launched in 1991, and the first integrated MEMS gyroscope was released in 2002. The newest MEMS products released by ADI are ADGM1304 and ADGM1004, the maximum operating frequency can reach up to 14 GHz and 13 GHz, respectively. The operation temperature ranges from 0 to 85 °C, with the peak reflow soldering temperature of 260 °C. In [22], it is shown that the SiC MEMS devices are well-developed for temperatures up to 500 °C for the sensing of motion acceleration and gas flow. The digital micro switches can also be used for wireless power transfer, but the maximum operating temperature has not been reported. Reference [88] reported nanoelectromechanical system switches based inverter can operate at the temperature as high as 500 °C with ultralow leakage current, and this achievement has created a pathway toward energy-efficient high-temperature computation. 

## 4. Challenges in High-Temperature Power Electronics

### 4.1. Design of High-temperature Gate Drives

Gate drives play an important role in the interface the control circuit to SiC-based devices, determining the performance of power electronics devices. Although SiC-based devices have high-speed switching capability, the drive circuit should also be matched to make full use of high-speed switching capability. Therefore, the SiC high-temperature drive circuit cannot follow the drive circuit based on conventional Si devices. Since the parasitic capacitance of a similarly-sized silicon carbide device is much lower than that of Si-based devices. A tradeoff should be taken between the component’s layout and the high-speed capability, so the drive circuit should be placed at a certain distance to the power electronic devices. This will increase the gate loop and introduce larger parasitic parameters, which decrease the high-speed switching capability of the SiC-based devices in practical application. Then, the switching frequency will be limited under this condition.

The performance of SiC power electronic devices might be degraded when employed in high-temperature condition. By testing, the threshold voltage of 1.2 kV SiC MOSFET devices at 200 °C is reduced to 2/3 under normal temperature, while the on-resistance increases to 2–3 times. The reduction of threshold voltage makes the crosstalk occur more easily in the bridge-arm circuit [89]. The method of gate negative bias voltage is adapted to suppress the crosstalk, in which the negative bias voltage usually arrives at −5 V and the minimum value is −9 V. Furthermore, the active miller clamping circuit is designed to avoid the bridge-arm shoot-through. On the other hand, the gate drive board is allowed for a high-temperature environment only if the SOI die, PCB, passive components, packaging, as well as the input signal isolator, can endure the high temperature. This is expected to reduce the volume of easy chip-level integration, eliminate the high-temperature ageing effects of materials, and reduce the impact of parasitic parameters.

### 4.2. Current Measurement in High Temperature

**Figure 17:**
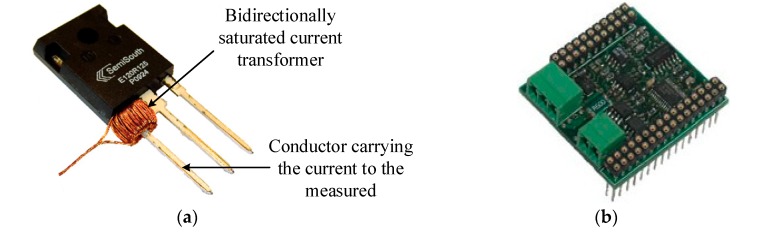
Fast high temperature isolated DC/AC current measurement. (**a**) Photograph of the bidirectional saturated current transformer, and (**b**) PCB circuit.

The current divider and Hall sensor are usually employed to measure the current for converter control; however, they are challenging to work in the high-temperature environment. To tackle this problem, the saturated current sensor is developed for high-temperature application. While the B/H curve of magnetic materials can drift with the temperature variation, which will lead to a significant measurement error for the current sensor, a compensation algorithm or new measurement method is proposed for the high measurement accuracy. For example, the isolated DC and AC current measurement method based on a bidirectional saturated current transformer (Figure 17) can be applied to the high-temperature converter with SiC devices, which can suppress the effect of the coercive force of magnetic materials on detection precision [90].

### 4.3. Parameters Matching within Wide Range of Temperature 

The parameters matching over a wide temperature range is also an issue to design the high-temperature power electronics. The performance of the SiC components will degrade under the wide temperature cycling and the high operating temperature. With the increase of the operating temperature, the junction capacitance of SiC devices is decreased, and the switching speed is increased. The performance of passive components, such as capacitors and resistors, is also reduced, and the withstand voltage, capacitance values, and resistance values are only about 40% to 50% of normal temperature. In addition, the coefficient of thermal expansion between adjacent components should be similar; otherwise excessive mechanical stress will cause damage to the device.

Since SiC material defects and brittleness limit the size of the wafer to a small value of 4 inches in general use, the maximum current of a single chip is about 100 A. The cost of SiC chip grows exponentially with the chip current. For the application of high-power converters, the multichip power module is a cost-efficiency solution. Among paralleled chips, the parameter mismatching due to the parasitic parameters can result in the uneven electro-thermal stress. For all kinds of active and passive components, their temperature stability difference can result in the unmatched electric parameters and unbalanced mechanical stress, which significantly influence the performance and reliability of high-temperature converters.

### 4.4. High-temperature Packaging Technology

SiC converters and MEMS devices face the challenge of a minimum of parasitic parameter and capability of operating in a high-temperature environment. Due to the fast switching speed and low threshold voltage, SiC devices are deeply affected by the inherent and line parasitic parameters, which requires the packaging design to minimize the length of pin and wire, but the compact layout reduces the area of dissipation [91]. Likewise, the packaging technology is also a challenge to the fabrication of SiC devices for high-temperature applications. The high-temperature welding technique is one of the critical factors to improve the high-temperature capability of SiC devices [92]. The advanced material technique and the innovative structure design need to be developed to approach the SiC physical limitation.

Packaging materials and structures with improved reliability at higher temperatures are imperative for the implementation of SiC devices, mentioning the high-temperature die attach, high-conductivity TIM, and aggressive heat rejection system. For the die attach, CTE, melting temperature, porosity feature, as well as electrical and thermal conductivity are not the only indices [93]. The mechanical properties such as the modulus of elasticity, ductility, and yield strength are of equal importance [94]. Lead-free gold-based solders are a good choice for niche applications, but their mechanical stiffness is as a limiting factor, which can transfer stresses to SiC devices resulting in die cracking. The thermal resistance of TIM is still the bottleneck in most power electronics packaging, so the high-performance filler materials should be further investigated as both an enhancement and a basis for TIMs. Double-sided cooling structures, two-phase cooling methods and novel coolants can improve the cooling capability for SiC modules, which need further demonstration and understanding of long-term reliability before industrial applications. 

## 5. Conclusions

To meet high requirements for industrial applications like the electric vehicle, electric aircraft, deep-earth oil and gas exploration, and geothermal energy development, developing the high-temperature power electronics with a significant increase of power density, efficiency, and reliability is indispensable. Compared with conventional Si power electronic devices, SiC power electronic devices have many advantages, including improved converter performance, reduced volume and weight, and simplified heat dissipation structure. This paper presented an overview of the development of high-temperature component profiles. It indicated that the advanced materials and technologies are vital for the performance promotion of high-temperature converters. Three typical application examples of SiC high-temperature converters and MEMS devices have been studied, which show the application status in several fields and uncover the main existing issues. Although many researchers are currently focusing on high-temperature packaging and integration technologies, high-temperature power electronics with SiC devices appear promising for reliable operation in a wide temperature range. Indeed, the performance of SiC converters and MEMS devices can be further enhanced when the high-temperature packaging and gate drive progress, and when the measurement and parameter matching problems are well resolved.

## Figures and Tables

**Table 1:** Applications of high-temperature power electronics.

High Temperature Application	Peak Ambient	Current Technology	Future Technology
Automotive			
Engine control electronics	150 °C	Bulk Si and SOI	Bulk Si and SOI
Electric/ Hybrid vehicle power management and distribution (PMAD)	150 °C	Bulk Si	WBG
Electric suspension and brakes	250 °C	Bulk Si	WBG
On-cylinder and exhaust pipe	850 °C	N/A	WBG
Turbine engine			
Sensors, telemetry, control	300 °C/600 °C	Bulk Si and SOI/N/A	WBG and SOI/WBG
Electric actuation	150 °C/600 °C	Bulk Si and SOI/N/A	WBG
Deep-well drilling telemetry			
Oil and gas	300 °C	SOI	WBG and SOI
Geothermal	600 °C	N/A	WBG
Industrial			
High-temperature processing	300 °C/600 °C	SOI/N/A	SOI/WBG
Spacecraft			
Power management	150 °C/500 °C	Bulk Si and SOI/N/A	WBG
Venus and Mercury exploration	550 °C	N/A	WBG

Note: Bulk Si, SOI, WBG, and N/A stand for bulk silicon, silicon-on-insulator, wide band-gap, and currently no available, respectively.

**Table 2:** The physical properties of the available semiconductor materials under room temperature (25 °C).

Items	SiC			Si	GaAs
	4H-SiC	6H-SiC	3C-SiC		
Band-gap (eV)	3.2	3.0	2.2	1.12	1.43
Maximum operation temperature (°C)	1580	1580	1580	600	400
Breakdown field strength (V/cm)	2.2 × 10^6^	2.5 × 10^6^	2.0 × 10^6^	0.3 × 10^6^	0.4 × 10^6^
Maximum electron saturation velocity (cm/s)	2.0 × 10^7^	2.0 × 10^7^	2.5 × 10^7^	1.0 × 10^7^	1.0 × 10^7^
Thermal conductivity (W/cm·K)	3~4	3~4	3~4	1.7	0.5
Electron mobility (cm^2^/s·V)	980	370	1000	1350	8500
Hole mobility (cm^2^/s·V)	120	80	40	480	400

**Table 3:** The thermal mechanical characteristics of materials for the substrate.

Ceramic Materials	96% Al (Al_2_O_3_)	99% Al (Al_2_O_3_)	AlN	BeO	Si_3_N_4_
Dielectric field intensity (kV/mm)	12	12	15	12	10
CTE (ppm/°C)	60	7.2	4.5	7.0	2.7
Thermal conductivity (W/m·K)	24	33	170	270	60
Structural strength (g/cm^3^)	3.5	4.0	2.6	4.0	5.0
Flexural strength (MPa)	317	345	360	250	850
Tensile strength (MPa)	127	207	310	230	17

**Table 4:** The thermal mechanical attributes of materials for the base plate.

Materials	100 Cu	100 Al	85 Mo/15 Cu	75 Mo/25 Cu	65 Mo/35 Cu
CTE (ppm/°C)	17.8	26.4	6.8	7.8	9.0
Thermal conductivity (W/m·K)	398	230	165	185	205
Density (g/cm_3_)	8.90	2.70	10.01	9.87	9.74
Young modulus (MPa)	128,000	70,000	274,000	274,000	274,000
Yield strength (MPa)	210	50	/	/	/

**Table 5:** The thermal mechanical characteristics of materials for die attach.

Solder	Sn96.5 Ag3.5	Sn95 Sb5	Pb75 In25	Sn10 Pb88 Ag2	Sn10 Pb90	Au20 Sn80
Melting point (kV/mm)	221	235	240	267	275	280
Boiling point (ppm/°C)	221	240	260	290	302	280
Density (W/m·K)	7.5	7.25	9.97	10.75	10.75	14.51
Thermal conductivity (W/m·K)	33	28	18	27	25	57
CTE (ppm/°C)	30	31	26	29	29	16
Electrical conductivity (S/m)	16	11.9	4.6	8.5	8.9	/

**Table 6:** Commercially manufactured capacitors for high-temperature applications.

Capacitor type	Packaging	Max. Temperature	Comments
Solid tantalum	SMT and through-hole	230 °C	High capacitance value; voltage degradation 60–80% at 230 °C
Electrolytic wet tantalum	Axial through-hole	200 °C	High capacitance value; most through-hole
Stacked ceramic	SMT-chip stacked gull-wing	260 °C	Poor stability above 175 °C for X7R, but higher capacitance density than C0G

**Table 7:** Commercially manufactured resistors for high-temperature applications.

Resistor Type	Packaging	Max. Temperature	Comments
Metal foil	Through-hole, surface-mount device (SMD)-chip, flip-chip	240 °C	High precision
Metal oxide	Through-hole	275 °C	General purpose, power resistors, good frequency characteristics
Thin film	SMD-chip, flip-chip	275 °C	Compact, low TC, high stability
Thick film	SMD-chip, flip-chip	300 °C	General purpose, wide resistance range
Wire-wound	Through-hole	350 °C	High surge capability, precision power
